# Self Help for Hospitals and Their Patients

**Published:** 1898-12-17

**Authors:** 


### The Institutional Workshop.

### SELF HELP FOR HOSPITALS AND THEIR PATIENTS.

The duty and the dignity of self help was the theme of Sir Arthur Arnold's discourse to the delegates of the Hospital Saturday Fund at the Mansion House the other day, and beyond question self help is a virtue in praise of which too much cannot be said. Hospitals are, of course, primarily charitable institutions, but it is the merest platitude to say that the condition of their finances would be far more satisfactory than is commonly the case, and their efficiency therefore greater, if something could be done towards making them, to some extent at least, self-supporting. "Grant that we need no help of men, that we may help such men as need," is an aspiration that should come from the heart of every hospital authority.

A suggestion with this end in view has been made which would also have another great advantage—that of inculcating self-help among the masses to whom it would be of the greatest service, and who, while constituting the chief users of the hospitals, give least toward their support. It is that hospitals should ask their out-patients—other than children—to contribute, according to their means, towards the cost of the benefits they are receiving, giving them in acknowledgment the stamps issued by the Prince of Wales's Fund; these to be supplied to the hospitals for the purpose free of charge.

This scheme would, of course, entail at first some amount of extra effort on the part of already hard-worked secretaries and dispensers, but "*absque labore nihil*," and the resulting income would soon far more than provide for the cost of the organisation involved, while the advantages of the plan when in full working order, and widely adopted, would be incalculable—would indeed put hospitals on an entirely fresh footing.

The Central London Throat and Ear Hospital has been experimentally trying this scheme in part, and has found it pay well. As is well known, this institution is one which for many years has been in the habit of taking from its out-patients contributions according to their means, so there has not been in this case the need for the organisation *ab initio* of the administrative details necessary; but the fact that in the couple of months or so the stamps have been in use a substantial addition to the income from this source has been made, shows that they do act as a stimulus to givers. There is something to show for the money—a record that one is a "subscriber"—and as such of distinct benefit to the self-respect of the contributor, and also to the revenue of the hospitals generally, since friends and neighbours, to whom the stamps are shown, get into their heads also the much-needed notion that hospitals don't "run themselves."

That this idea wants bringing home to the working-classes is well illustrated by the experience of the Poplar and the London Hospitals. For years the subscription boxes at their doors scarce yielded enough to pay for keeping them there. But when these hospitals instituted a small charge to out-patients for medicines and bandages, money began to be dropped into the boxes also, and each year shows an increase in the amount so collected.

The field is a vast one, and practically untouched, for only a very small fraction of the seven millions and more within the metropolitan area give anything toward the support of the hospitals, except in the most casual and intermittent fashion. The idea of the stamps is to create a very large number of regular annual subscribers of small amounts, and if each family within the district directly benefited by the London hospitals subscribed but a penny a month—say a farthing a head—and so bought one shilling stamp per annum, the hospitals would get well over £100,000 a year, and so be put on a sound and satisfactory basis.

The Saturday Fund also can do much toward popularising the stamps and familiarising people with their use. The Council of the Prince of Wales's Fund are willing to supply the stamps to firms where workshop collections are made free of cost, so that they may be used as an extra inducement to get workpeople to add their names to the subscription lists. Hitherto these men have had no credit, as individuals, for the amounts they have contributed. But under the proposed arrangement, if they each buy one of the attractive little stamp albums issued by the Prince's Fund, to be obtained at most booksellers at a cost of 6d. each, and which will last for many years, they will have evidence in their own hands that they are regular subscribers to the support of the hospitals. And in addition, by this means, a hitherto almost untouched class may be tapped—the many thrifty artisans in business for themselves outside the big establishments, and also small tradespeople. The stamps constitute a handy form of receipt, and give the holder a personal and permanent interest in hospital work in general, which is bound to bear good fruit.

